# Intracranial pressure monitoring in acute liver failure: a retrospective cohort study

**DOI:** 10.1186/cc10915

**Published:** 2012-03-20

**Authors:** C Karvellas, O Fix, H Battenhouse, V Durkalski, C Sanders, W Lee

**Affiliations:** 1 University of Alberta, Edmonton, Canada; 2 UCSF, San Francisco, CA, USA; 3 Medical University of South Carolina, Charleston, SC, USA; 4 University of Texas-Southwestern, Dallas, TX, USA

## Introduction

Intracranial hypertension (ICH) complicates roughly 25% of acute liver failure (ALF) patients with grade III/IV encephalopathy. Intracranial pressure (ICP) monitoring is controversial due to complications in 5 to 20% and absence of documented mortality benefit.

## Methods

Using prospectively collected data from the US Acute Liver Study Group registry, we reviewed 630 ALF patients with severe encephalopathy (grade III/IV) and INR >1.5 enrolled between 1 March 2004 through 31 August 2011. ICP monitoring was used in 143 patients (23%); 487 control patients with grade III/IV hepatic coma (*n *= 487) were not monitored.

## Results

The most common etiology of ALF was acetaminophen (51%, *P *= 0.13 between groups). Of ICP monitored (ICPM) patients, 85% (*n *= 121) received devices within 24 hours of admission to study. ICPM patients were significantly younger (36 ± 6 years vs. 43 ± 15 years, *P *< 0.001) than controls, more likely to be on renal replacement therapy (48% vs. 31%, *P *< 0.001) but less likely to be on vasopressors (20% vs. 32%, *P *= 0.008). ICPM patients were given more ICH directed therapies (mannitol 43% vs. 13%, hypertonic saline 21% vs. 6%, hypothermia 29% vs. 11%, *P *< 0.001 for each comparison). For ICPM patients, the median INR on the day of monitor insertion was 2.2 (1.6 to 2.9) and platelet count 116 (84 to 171); 74% were given FFP (vs. 46% controls, *P *< 0.001) and 19% (vs. 14% controls, *P *= 0.14) received platelets. ICP monitoring was also strongly associated with listing (78% vs. 27%, *P *< 0.001) and receipt of liver transplant (42% vs. 18%, *P *< 0.001). Twenty-one-day mortality was similar between ICPM patients (33%) and controls (37%, *P *= 0.33) when all or only nontransplanted patients (46% vs. 45%, 0.8) were considered. Of 66 ICPM patients with detailed information, 18 (29%) had evidence of ICH (ICP >25 mmHg) at the time of ICPM insertion (maximum ICP on day 1 ~18 (12 to 26) mmHg). Of 49 patients with a known ICPM device, 14 patients received epidural catheters, six subdural, 11 intraparenchymal, seven intraventricular and 11 lumbar monitors. In only one of 49 ICPM patients was intracranial hemorrhage reported, and this patient survived.

## Conclusion

In ALF patients, ICP monitor placement is strongly associated with liver transplantation but not with overall or transplant free mortality. In the absence of ICP monitoring, ALF patients may be less aggressively treated for intracranial hypertension. The value of ICP monitoring in ALF remains to be determined but ICPM placement clearly affects the frequency of interventions for elevated ICP.

